# Biomechanics of the finger pad in response to torsion

**DOI:** 10.1098/rsif.2022.0809

**Published:** 2023-04-19

**Authors:** Sophie du Bois de Dunilac, David Córdova Bulens, Philippe Lefèvre, Stephen J. Redmond, Benoit P. Delhaye

**Affiliations:** ^1^ School of Electrical and Electronic Engineering, University College Dublin, Belfield, Dublin 4, Ireland; ^2^ Institute of Information and Communication Technologies, Electronics and Applied Mathematics (ICTEAM), and Institute of Neuroscience (IoNS), Université catholique de Louvain, 1348 Louvain-la-Neuve and 1200 Brussels, Belgium

**Keywords:** skin mechanics, touch, torque, slip, finger

## Abstract

Surface skin deformation of the finger pad during partial slippage at finger–object interfaces elicits firing of the tactile sensory afferents. A torque around the contact normal is often present during object manipulation, which can cause partial rotational slippage. Until now, studies of surface skin deformation have used stimuli sliding rectilinearly and tangentially to the skin. Here, we study surface skin dynamics under pure torsion of the right index finger of seven adult participants (four males). A custom robotic platform stimulated the finger pad with a flat clean glass surface, controlling the normal forces and rotation speeds applied while monitoring the contact interface using optical imaging. We tested normal forces between 0.5 N and 10 N at a fixed angular velocity of 20° s^−1^ and angular velocities between 5° s^−1^ and 100° s^−1^ at a fixed normal force of 2 N. We observe the characteristic pattern by which partial slips develop, starting at the periphery of the contact and propagating towards its centre, and the resulting surface strains. The 20-fold range of normal forces and angular velocities used highlights the effect of those parameters on the resulting torque and skin strains. Increasing normal force increases the contact area, the generated torque, strains and the twist angle required to reach full slip. On the other hand, increasing angular velocity causes more loss of contact at the periphery and higher strain rates (although it has no impact on resulting strains after the full rotation). We also discuss the surprisingly large inter-individual variability in skin biomechanics, notably observed in the twist angle the stimulus needs to rotate before reaching full slip.

## 1. Introduction

Humans are incredibly skilled at manipulating objects. Our success at dexterous manipulation relies on the feedback from our sensory systems, in particular tactile, proprioceptive and visual. Amongst them, the contribution of tactile feedback is essential, in that removing it by trauma or anaesthesia of the fingers leads to major performance degradation even in the simplest manipulation tasks [1,2]. Moreover, cutaneous feedback influences weight perception, and subsequently, the grip force used during object manipulation [3].

What we sense by touch is mediated by the mechanoreceptors, the sensory endings of the afferent nerves densely innervating the skin [4], which transduce mechanical deformations at the level of their end-organs into neural activity [5]. Indeed, mechanical events that fail to induce sufficient deformation of the skin cannot be sensed [6,7]. Knowing how skin deforms under loading is a crucial step in understanding how our sense of touch is generated and subsequently used to guide our actions in tasks such as object manipulation [8].

While mechanoreceptors transduce local deformation of the skin, it is the integration of the information coming from numerous tactile fibres over a larger area of skin that enables us to decode the properties of the interface between the skin and the object being manipulated [9,10], such as normal and shear forces, slip, texture, friction and torque, for example [11–15]. Through further inference, the properties of the object itself may be estimated, e.g. its mass. To better understand the nature of this population-level tactile information, it is therefore relevant to study how the skin of the finger pad deforms when subjected to mechanical stimuli [16–18].

The contact interface between a passive finger (i.e. the participant is relaxed and does not attempt to move the finger) and a moving glass plate has been studied previously. When rectilinear tangential loads are applied to the finger pad, the periphery of the contact area between the skin and the glass plate starts slipping first. This slip annulus then propagates towards the centre of the contact area until all skin in contact with the glass plate is slipping [19]. Surface strains are present at the border between the stuck area and the slip annulus [20], and can be linked with FA-I firing rates [21]. The partial slip phase of the episode is of particular interest, as it might give us an interval of time during which we can sense the impending full slip and so adjust our grip force before the object in our hand starts to escape our grasp [22,23].

Most studies looking at the evolution of the slip annulus have focused on rectilinear motion [19–21,24–26]. However, in the likely event that the lifting force vector applied by the fingers to the object does not pass through the object’s centre of mass, the weight of the object may produce a net torque around the point of contact. This torque can be counteracted by a reactive torque arising from the friction at the finger–object interface, which can be modulated by the normal force applied. [27]; for a two-finger precision grasp, there are of course two finger–object interfaces [28], but the idea remains the same. The minimum grip force required to prevent slip arises from the resultant combination of tangential forces and torques [27,28]. In addition, the minimum grip force depends on the frictional condition [27] and the curvature of the grasped surface [28], in response to which participants can appropriately scale their grip force.

Cutaneous feedback elicited by torsion contains information about the applied torque. Passive experiments in monkey [29] and human fingers [15] showed that the magnitude and direction of the torque applied by a flat stimulus could be extracted from the population response of sensory afferents, despite their complex responses at an individual level. On the basis of the link established between surface skin strains and neural recordings in rectilinear motion [21], we expect that some of the inter-afferent variability observed under torsion might be explained by specific local strains present in their receptive fields. Overall, understanding how skin deforms locally under torsion could help uncover unknown properties of the mechanotransduction process, and in a wider perspective, how humans scale their grip force in the presence of torques.

To date, and to our knowledge, there have been no studies on the mechanics of partial slip on the finger pad arising from torsion. This study is a first step towards understanding cutaneous feedback in the presence of torque. Here, we extend the work studying the dynamics of the finger pad under translation [20,26,30] to rotations. To that end, we passively stimulated the finger pad with a flat transparent surface, under controlled normal forces and rotation speeds, both of which were varied to span a range relevant to human touch in everyday scenarios, while monitoring the contact interface using optical imaging. We report the characteristic pattern by which the slip develops, the strong influence of force and speed and the large inter-individual variability that suggests exercising caution when analysing data coming from multiple participants.

## 2. Methods

### 2.1. Participants

Seven healthy human volunteers (aged 23–35, 4 males) participated in the study. All provided informed consent before participating in this study. The experimental procedure was approved by the local ethics committee at the Université catholique de Louvain.

### 2.2. Experimental protocol

**Figure 1.**
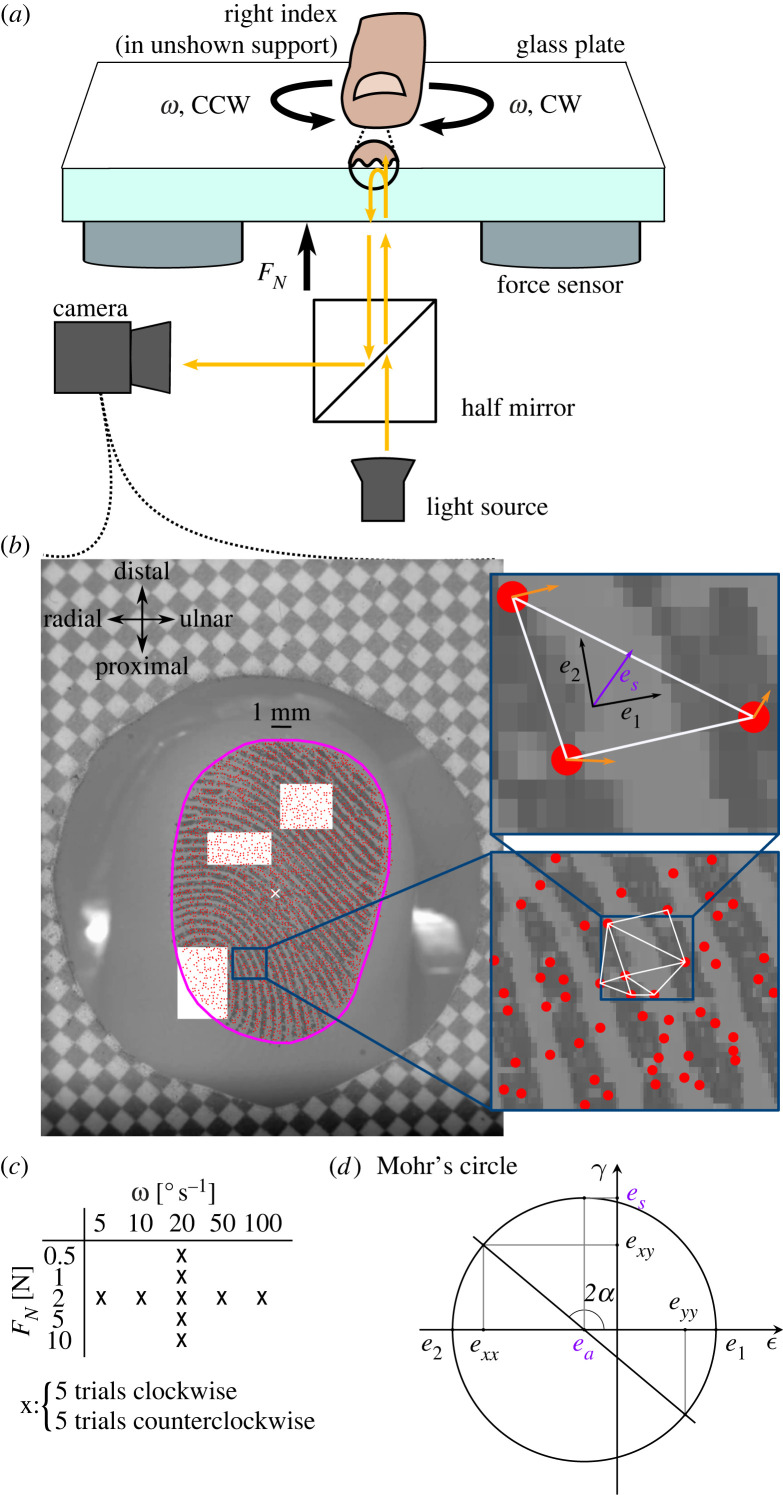
Methods. (*a*) Participants rested the nail of their right index finger in a support, volar side pointing down. Below, a robot controlled the position of a smooth glass plate. The normal force applied was measured by two force and torque sensors located between the robot and the plate. The finger–plate contact was illuminated and filmed through the glass plate. (*b*) Video frame acquired by the apparatus. The border of the contact is shown in magenta. Features of the contact were tracked (red dots) and formed the vertices of a Delaunay mesh (white triangle). Areas of skin slipping and Green–Lagrange strains were derived from the skin displacement field (orange arrows). (*c*) Comparisons across normal forces were made for a fixed angular velocity (20° s^−1^), and comparisons across angular velocities for a fixed normal force (2 N). (*d*) Mohr’s circle is a graphical representation of the strain tensor in multiple coordinate systems. In Mohr-circle space, the abscissa and ordinate represent, respectively, the normal and shear strain. Each point on the circle represents the strain tensor in a given coordinate system obtained by rotation. A rotation of *α* in the physical world corresponds to a rotation of 2*α* in Mohr-circle space. The two intersections between the circle and the abscissa represent principal strains (absence of shear strain). The radius of the circle is the maximum shear strain, obtained in the coordinate system at 45° from the principal strains (90° in Mohr-circle space). The circle centre represents the average magnitude of the two principal strains or area invariant.

A platform consisting of an industrial four-axis robot (DENSO HS4535G), two six-axis force sensors (ATI Industrial Automation, Mini 40), a glass plate, a finger holder and an imaging system was used to perform the experiment (figure 1*a*). More details about the setup can be found in the studies by Delhaye *et al.* [20,26]. Because of the angle of the fingerpad relative to the plate, the initial contact generates a non-null tangential force component. If the contact is not exactly centred on the force sensors, this will also lead to a measured torque. We made sure that this torque was as small as possible by adjusting the platform’s horizontal position with respect to the finger such that it would generate minimal torques after loading. During a trial, the plate was brought into contact with the participant’s right index finger until the desired normal force level (*F*_*N*_) was reached. After a 1 s pause, the robot rotated the plate at a constant angular velocity (*ω*) until a rotation of 80° was reached, and then stayed at that position for 1 s. The glass plate was cleaned with a microfiber cloth between trials. Five different normal force levels were tested (0.5, 1, 2, 5, 10 N) at an angular velocity of 20° s^−1^, and five different angular velocities were tested (5, 10, 20, 50, 100° s^−1^) at a normal force of 2 N (figure 1*c*); thus, a 20-fold range for both parameters. As the camera focus had to be adjusted for each normal force level, trials were presented in the fixed order: 0.5, 1, 5, 10 and 2 N. Then at 2 N, the order of presentation of angular velocities was pseudo randomized. Each condition was repeated five times in clockwise (CW) and counterclockwise (CCW) directions, for a total of 90 trials. The same glass plate was used for all participants and trials.

Images of the finger pad were captured using a camera (JAI-GO-5000M-PMCL) with a resolution of 2560×2048 pixels (leading to a resolution of 85 pixels mm^−1^) and a frame rate equal to the target angular velocity in ° s^−1^ (5–100 fps); see figure 1*b*. The finger–plate contact was illuminated by a diffuse light source and filmed through the glass plate. Forces, position and orientation data from the robot were sampled at 1 kHz.

#### 2.2.1. Precise measurement of torque

**Figure 2.**
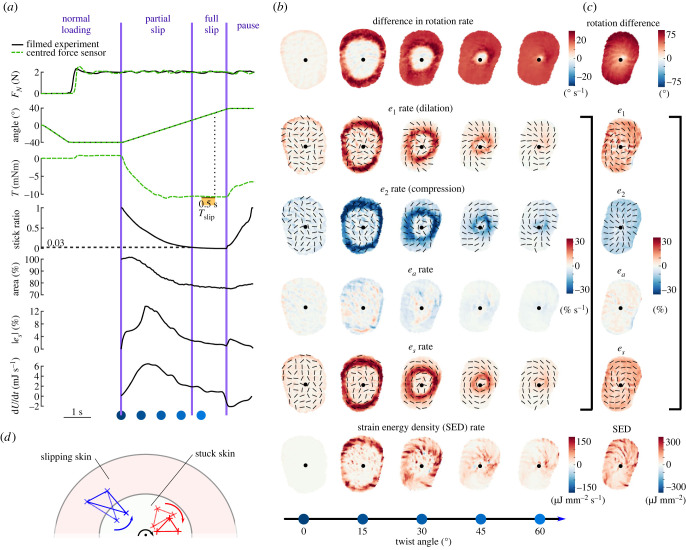
Illustrative trial. Data come from both a trial with video recording and a corresponding trial with precise torque measurements, at 2 N, 20° s^−1^, counterclockwise, of Participant 2. (*a*) Time evolution of normal force, stimulus angle, torque, stick ratio, contact area, median of the maximum shear strain over the contact area and strain energy for an illustrative trial. Black traces come from one trial of the experiment with video recordings, while green traces come from a corresponding trial of the experiment measuring torque precisely with a force sensor directly under the finger. The horizontal dashed line on the stick ratio plot highlights the time and angle when full slip occurs. Blue dots correspond to the frames displayed in (*b*). (*b*) Various descriptions of the skin strain rates in rows, with columns showing snapshots at times represented by the blue dots in (*a*) throughout plate rotation. The centre of rotation is marked with a black dot. *Rotation rate* of the skin is expressed in the frame of reference of the rotating plate, with the increasing intensity of colour indicating a greater difference in rotation rates of skin and plate, implying that the skin is slipping. *Two principal strain rates* (*e*_1_ and *e*_2_) express the local deformation as purely dilation and compression (i.e. the local reference frame is chosen so that the shear component is zero), and associated eigenvectors are indicated by black lines. *Area strain invariant* (*e*_*a*_) describes the local change in skin area. The *maximum shear rate* (*e*_*s*_) is determined by choosing the local coordinate frame such that the shear component of the local strains is maximized, at 45° from *e*_1_ and *e*_2_. Finally, the *strain energy density* is shown, which approximates the energy stored in the skin surface due to local deformations, assuming a Young’s modulus of 1 MPa and a Poisson ratio of 0.4. (*c*) The rightmost column shows the delta of the corresponding row variable between the last and first frames of the 80° rotation. (*d*) Schematic view of the finger with a region of the skin slipping and a region of the skin stuck to the plate. The area inside the large red oval represents the contact area, and the area inside the small white oval represents the skin stuck to the glass. The red triangle represents a triangular skin element with all its vertices inside the stuck part of the skin, and the blue triangle represents a triangular skin element with two of its vertices in the part of the skin slipping and one vertex in the part of the skin stuck to the plate. The black dot indicates the centre of rotation, and the black arrow indicates the rotation direction of the plate. The difference in the rotation rate between the red triangle and the plate is 0, while it is greater than *ω* between the blue triangle and the plate.

**Figure 3.**
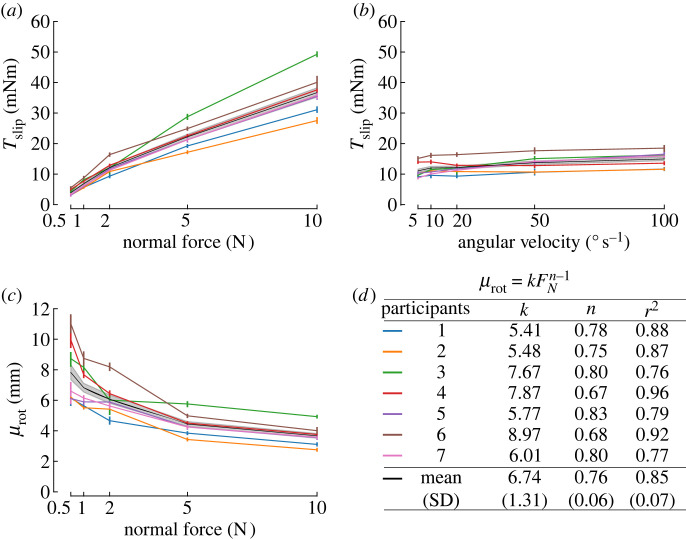
Evolution of torque plateau (*T*_slip_) with normal force and angular velocity, and evolution of the rotational coefficient of friction with normal force. In all panels, the black line is the mean across participants, with corresponding 95% confidence intervals in shaded grey. Individual participants are presented as light-coloured lines, with colours defined in (*d*). (*a*) Maximum torque for all normal forces tested. (*b*) Maximum torque for all angular velocities tested. (*c*) Coefficient of rotational friction *μ*_*rot*_ as a function of normal force (*F*_*N*_). (*d*) Least-squares fit of the coefficient of rotational friction as a function of the normal force as the power law: $\mu_{\mathrm{rot}}=kF_{N}^{n-1}$.

After collecting the data from the first experiment, we realized that the tangential component of the force measured by one of the two sensors was corrupted, precluding the torque estimation from the collected dataset. Nevertheless, we thoroughly tested and verified that the normal component of the force recorded was still valid and therefore usable. Then, we had the choice of re-running the same experiment again to obtain a measure of the torques, or running a separate experiment with a more precise measurement of the torque, given the low signal-to-noise ratio (SNR) resulting from the mounting configuration. Indeed, given that the torques we measure are of the order of tens of mNm (see figure 3*a*,*b* for the range relevant to our study), the SNR for both mounting configurations can be estimated as follows: In the first experiment, the torque is evaluated by the difference of two separate tangential force measurements each made 10 cm away from the centre of the plate (approximately where the finger pad contact is centred). Therefore, taking as example a torque of 10 mN m (expected for a trial at 2 N and 20° s^−1^), the expected tangential force measured at 0.1 m from the centre of rotation is around 0.1 N ( = 0.01 N m/0.1 m), which would be divided between the two sensors. This value is not much larger than the noise level (based on the exemplar trial illustrated in figure 2, where the standard deviation of the tangential force reading when the sensor is unloaded is 0.0069 N), leading to a poor SNR of 17.20 dB ( = 20 log_10_(0.05/0.0069)). In the second experiment, using a single centred force/torque sensor, the noise level of the torque reading is 0.087 mN m (average standard deviation of torque when the sensor is unloaded) for a signal of 10 mN m, leading to a much better SNR of 41.21 dB. Given the difference in SNR, we chose a more precise measurement of the torque, even though we did not have the concurrent imaging of the finger pad. Again, each trial was repeated five times, and all comparisons between the two experiments were performed on averages across those repetitions.

### 2.3. Data analysis

#### 2.3.1. Pre-processing

Force and torque data were filtered using a zero-phase Butterworth filter of order 2, with a cut-off frequency of 5 Hz.

#### 2.3.2. Torque

To study the effect of normal force and angular velocity on the torque generated by the rotation of the plate, we extracted the magnitude of the torque during its plateau (*T*_slip_) as the average value on the 0.5 s window preceding a twist angle of 75° (figure 2*a*). This angle was selected instead of 80° to avoid the deceleration phase of the plate.

#### 2.3.3. Coefficient of kinetic rotational friction

We computed the coefficient of rotational friction (*μ*_rot_), analogous to the coefficient of linear friction for each trial. The coefficient of rotational friction is defined in equation (2.1):

2.1 $$\mu_{\mathrm{rot}}=\frac{T_{\mathrm{slip}}}{F_{N}},$$

where *T*_slip_ and *F*_*N*_ represent the torque and the normal force during steady slip, respectively.

#### 2.3.4. Video processing

The checkerboard grid (figure 1*b*) was used to verify that image distortion was negligible. The frames of the video of the fingerpad area were filtered spatially with a Gaussian bandpass filter in the range of fingerprint ridges (0.4 mm; see [31]). Optimal features were detected and tracked from frame to frame using the Lucas–Kanade–Tomasi algorithm implemented in the OpenCV toolbox [32–34], and used to compute the skin displacement field. The displacement field was filtered using a convolution with a Gaussian window (*N* = 16, σ=0.75 frames). A Delaunay triangulation of those features was computed, tessellating the contact area into triangular skin elements (figure 1*b*).

#### 2.3.5. Apparent contact area

The apparent contact area *A*^*A*^ was segmented using a semi-automatic machine learning algorithm (see the magenta border in figure 1*b*). The size of the contact area was determined by counting the pixels inside the segmented region and by converting the result to square millimetres using the conversion ratio of 85 px mm^−1^ given by the paper checkerboard pattern stuck on the glass and visible in every frame (figure 1*b*). The relative change of orientation of the contact area was obtained by computing the difference between its tilt at initial contact and the tilt when fully slipping. The tilt of the contact area was approximated by the angle between the *y*-axis of the camera’s reference frame and the major axis of an ellipse fitted on the contour of the contact area following the same procedure as in the study by Delhaye *et al.* [26].

During the stick-to-slip transition, the contact area can be modified by two mechanisms: skin breaking or making contact, and surface deformations.

To quantify the area of skin lifting off or entering contact at the contact edges (respectively, peeling or laying; see also [35] for terminology), we summed the areas of all Delaunay triangles entering or leaving contact. For a triangle to be considered in the contact area, its three vertices had to be located inside the segmented contacted area. Areas of triangles passing from inside to outside of the contact counted towards skin peeling. The Delaunay triangles were small (0.022 mm^2^ on average) with respect to the contact area (125.94 mm^2^ on average), ensuring a good resolution for the peeling and laying estimates.

To compute the influence of surface deformations on area change, we studied the frame-to-frame area ratio of all Delaunay triangles in the segmented contact area.

#### 2.3.6. Propagation of slip

To measure the amount of skin slipping on each frame of the video, we considered that a triangular skin element had slipped if its rate of rotation differed from that of the rigid plate by more than 0.6° per frame. The rate of rotation of a triangle was defined as the average angular displacement of the triangle vertices around its centroid. We then computed the stick ratio as the ratio of the area of skin stuck to the plate on a frame over the total area of skin in contact with the glass plate. The moment of full slip was defined as the moment when the stick ratio becomes smaller than 0.03, as slip is challenging to detect close to the rotation centre where the displacement of the stimulus is almost zero.

The border between the slipping and stuck skin was extracted by interpolating the location of the slipping skin on a regular grid and then by using Matlab’s function boundaries on the largest connected component.

#### 2.3.7. Local strains

Two-dimensional Green–Lagrange strains were computed at the skin–plate interface by computing the change in the shape of each triangular element between one frame and the next. The deformations of each triangular element are therefore quantified by a strain tensor ϵ having three independent values: the normal strain along the *x*-axis ($\epsilon_{xx}$), the normal strain along the *y*-axis ($\epsilon_{yy}$) and the shear strain ($\epsilon_{xy}$). The complete details of the strain computation can be found in [20].

We found that using a single coordinate system common to all triangular elements did not yield intuitive results. While the problem at hand involves rotations making a Cartesian system unappealing, it lacks rotational symmetry (elliptical shape of the contact area and potentially the pressure distribution not being symmetrical by rotation) which would enable the use of a polar coordinate system. Instead, we expressed strains in local Cartesian coordinates, specific to each triangular skin element at each frame, and obtained by rotations based on the direction of the principal strains.

Mohr’s circle, presented in figure 1*d*, is a graphical representation of the local strain tensor in all of such rotated coordinate systems and provides a graphical intuition for the following paragraphs.

The local principal strains express the local strains in a coordinate system which removes the shear strain component. For each triangular element and between each consecutive pair of frames, it is possible to find a set of perpendicular axes where the shear strain is zero. The strains along both axes are, respectively, the maximum and minimum strains across all orthonormal coordinate systems obtainable by rotation. Those are obtained by eigenvalue decomposition of the strain tensor ϵ. The obtained strains are called principal strains and named *e*_1_ and *e*_2_, respectively. This allows us to express the local deformation of the skin as a stretch along one local axis (maximum strain, *e*_1_ > 0) and compression along the perpendicular axis (minimum strain, *e*_2_ < 0).

From the principal strains, we can compute the change in the area of each triangular element (*e*_*a*_) as follows:

2.2 $$e_{a}=\frac{\left(e_{1}+e_{2}\right)}{2},$$

which also corresponds to the centre of Mohr’s circle (figure 1*d*).

The rotation of the plate in contact with the finger will likely mainly cause shear strains to happen on the skin. Therefore, we computed the maximum shear strain on each triangular element as follows:

2.3 $$e_{s}=\frac{\left(e_{1}-e_{2}\right)}{2},$$

which is the radius of Mohr’s circle (figure 1*d*).

To summarize and compare shear strain across conditions, we defined |*e*_*s*_| as the median of the maximum shear strain over the contact area and |*e*_*s*,end_| as the median of the maximum shear strain over the contact area after the full rotation.

#### 2.3.8. Strain energy

To estimate strain energy, which is the energy stored in the skin surface as it is deformed during the rotation, Young’s modulus and Poisson ratio were chosen to match the values estimated by [20], which are *E* = 1 MPa and *ν* = 0.4, respectively. The strain energy density (SED) *u*_*d*_ was obtained for each triangle element according to the following formula:

2.4 $$\begin{array}{rl} u_{d} & =\frac{E(1-\nu )}{2(1+\nu )(1-2\nu )}(\epsilon_{xx}^{2}+\epsilon_{yy}^{2})\\ & \;+\frac{E\nu }{\left(1+\nu )(1-2\nu \right)}\epsilon_{xx}\epsilon_{yy}+\frac{E}{\left(1+\nu \right)}{\epsilon_{xy}}^{2}. \end{array}$$

The surface SED was computed assuming homogeneous deformation over a depth *p* of 2 mm, similar to the study by Delhaye *et al.* [20] and in both cases chosen arbitrarily. This choice strongly affects the resulting SED, which we were cautious to only relate to other measures having made this same assumption about depth. The total energy over the contact area was evaluated as the sum of the triangles' energy weighted by their areas (*A*) following (2.5):

2.5 $$U=\int u_{d}\;dV≃p\int u_{d}\;dS\approx p\sum_{\begin{array}{c} i=1 \end{array}}^{T}u_{d}^{i}.A^{i}.$$

### 2.4. Statistical analysis

The influence of normal force, angular velocity and rotation direction were assessed with repeated measures analyses of variance (rANOVAs). The five repetitions of each condition were averaged. Because of the experimental design (holding force constant and varying angular velocity, or vice versa; cf. figure 1*c*), two rANOVAs were used per dependent variable. One used normal force as an independent variable, the other used angular velocity and rotation direction was included in both as a second independent variable. Mauchly’s test was used to test for sphericity. Greenhouse–Geisser correction was applied if sphericity was violated. Effect sizes were measured using partial eta squared (*η*^2^ = *SS*_effect_/(*SS*_effect_ + *SS*_error_)). Pairwise *t*-tests with Bonferroni correction for multiple comparisons were used for *post hoc* comparisons when rANOVAs showed a significant effect of one or multiple independent variables. The significance threshold used was 0.05.

## 3. Results

### 3.1. Illustration of a single trial run

An overview of the collected data is presented in figure 2, in an example trial from a typical participant at the centre of the parameter space. In this example, the normal force was set to 2 N and the angular velocity was 20° s^−1^ (see top two graphs in figure 2*a*). These typical traces show that, while the normal force was servo-controlled at a constant value, the plate was rotated a total of 80° at a constant speed for a total duration of 4 s.

As the plate started rotating, the friction between the glass and the finger skin generated a torque in the direction opposing motion (see torque *T* in figure 2*a*). This torque increased as the plate was rotated until it reached a plateau and remained stable for the rest of the rotation, at around −10 mN m in this example.

During the rotation, the transition from a fully stuck state to a fully slipping state occurs; i.e. the stick ratio transitions from 1 to 0 over the course of the rotation. The progression of the slipping front can be very clearly observed in a heat map showing the difference in rotation rate between the skin and the plate (see row ‘difference in rotation rate’ of figure 2*b*); when the skin is slipping, it is no longer rotating at the same angular velocity as the plate. Immediately outside of the stuck region, and travelling immediately behind the propagating slip-stuck boundary, the difference in rotation rate is higher than the angular velocity of the plate. In the fixed frame of reference of the room, the skin which has recently started slipping is stationary, while the skin stuck to the plate still rotates as a rigid body. A patch of skin overlapping those two regions rotates in the opposite direction as the plate, making their difference in rotation rate higher than the stimulus’s (figure 2*d*). As the periphery of the contact area, which slipped sometime before, becomes relatively immobile throughout the remainder of the trial, the twist happening closer to the centre of the contact area is compensated by this counter-twist (Stokes’ theorem). The slipping front progresses towards the centre of rotation, propagating as a wavefront, and reaches the centre at the moment of full slip. Along this wavefront, we observe the most extreme values of the skin deformation rate, which can be expressed in terms of either principal strain or maximum shear strain (see rows *e*_1_, *e*_2_ and *e*_*s*_ in figure 2*b*).

The principal strains, which express local deformation as purely dilation and compression, in a local coordinate system where there is no shear, are shown in the ‘*e*_1_ rate (dilation)’ and ‘*e*_2_ rate (compression)’ rows of figure 2*b*. Both *e*_1_ and *e*_2_ are maximal just behind the slip wavefront, with which their orientation forms a 45° angle. The skin behind the wavefront is slipping on the plate and not rotating at the same rate as the skin still stuck to the plate in front of the slip wavefront, giving rise to those strains.

We also observed in figure 2*a* (‘area’ subplot) that the apparent contact area decreases during the transition from fully stuck to fully slipping, similar to what is observed when the plate moves under linear tangential motion in the human finger [26] and elastomers [36]. This reduction in area cannot be solely explained by compression at the surface of the skin. When looking at the area invariant *e*_*a*_ in figure 2*b*,*c*, there is both compression and dilation at work. Those changes in area caused by dilation and compression are distributed non-homogeneously over the contact area. When looking at both *e*_*a*_ rate and the total *e*_*a*_ after the full rotation is complete, small wrinkles are present. As *e*_1_ and *e*_2_ (*e*_1_ and *e*_2_ lines of figure 2) are similar in magnitude but opposite in sign, they approximately cancel each other out, leaving the residual wrinkle pattern, as observed. As determined by visual inspection, these wrinkles do not seem to align with the fingerprint ridges. They represent small changes in area relative to the magnitude of deformation seen in the two principal strain components.

When using the local reference frame which maximizes the shear component of the local deformation (at 45° of the orientations of the principal strains), we also see an intense pattern of local shear along the slip wavefront. The orientations of the principal strains *e*_1_ and *e*_2_ are at 45° to the slip wavefront, so the shear *e*_*s*_ is maximal along the radial or tangential orientations (see black orientations in figure 2*b*, for *e*_1_, *e*_2_ and *e*_*s*_). After the full rotation, *e*_*s*_ is homogeneous over the contact area (see *e*_*s*_ row in figure 2*c*). Considering both the two principal strain variables and the maximum shear variable together, we see an overall picture which is dominated by shearing of the skin along the slip wavefront. Given the small overall change in local area (*e*_*a*_), the local strains are well summarized by the local maximum shear (*e*_*s*_) and its orientation. Therefore, the subsequent heat maps (figure 7) only show these two elements.

The rate of change of the total energy over the contact area typically followed a bell-shaped curve, starting at 0 mJ s^−1^ at movement onset, peaking at the middle of the partial slip phase and decreasing to a low, but non-zero, value at full slip (full slip does not imply homogeneous velocity field).

When the stimulus stopped rotating, elasticity of the skin made it rebound in the direction opposed to that of the stimulus. The torque reduced following this relaxation, and the contact area increased again. Negative strain energy rate is also observed, as the skin loses elastic energy. Visual inspection of the contact area traces suggests that the 1 s pause after each rotation is not sufficient to allow the skin to relax completely, as evidenced by the contact area increase appearing to still be in the early stages of an exponential relaxation pattern.

### 3.2. Torque and coefficient of rotational friction

Across all the normal forces applied (0.5 to 10 N), the torque plateau *T*_slip_ reached values of the order of tens of mN m. As expected, the maximum torque reached during the rotation increased with the normal force, with average values increasing from 3.91 mN m for a normal force of 0.5 N up to 36.70 mN m for a normal force of 10 N (figure 3*a*). The rANOVA showed a significant effect of normal force (*F* = 159.3, *p* < 0.001, *η*^2^ = 0.96) but not of the rotation direction (*F* = 0.02, *p* = 0.89). *Post hoc* tests between all pairs of *F*_*N*_ levels were significant.

Maximum torque also increased with angular velocity, but at a lower rate than with the normal force comparing figure 3*a*,*b*. Torques increased from a value of 11.06 mN m at an angular velocity of 5° s^−1^ to a value of 14.96 mN m at 100° s^−1^ (figure 3*b*). The rANOVA showed that there was a statistically significant effect of *ω* on *T*_slip_ (*F* = 9.90, *p* = 0.017, *η*^2^ = 0.26), but the effect size was smaller than for the normal force. There was no significant effect of the rotation direction (*F* = 0.015, *p* = 0.91). Amongst the *post hoc* pairwise comparisons, only the pair 50–100° s^−1^ reached significance.

By using the available torque and force data, we could also compute the coefficient of rotational friction *μ*_rot_ as the ratio of torque over the normal force during full slip. The coefficient of rotational friction between the plate and the participants’ finger pad varied significantly with changes in normal force, ranging from an average of 8 mm at 0.5 N of normal force to an average of 4 mm at 10 N of normal force (figure 3*c*). For comparison, the range of *μ*_rot_ reported by Kinoshita *et al.* spanned from 3.05 mm for rayon to 10.11 mm for sandpaper [27], which is comparable to the range of frictions we observed. Note, in their study, no dependency of *μ*_rot_ on *F*_*N*_ was visible for rayon, suede and sandpaper.

We observed that the coefficient of rotational friction decreased nonlinearly with normal force [37]. A negative power law, $\mu_{\mathrm{rot}}=kF_{N}^{n-1}$, was fitted to data of individual participants to capture the relation between *μ*_rot_ and *F*_*N*_. Across participants, the parameter *k* was equal to 6.74 ± 1.31 (mean ± standard deviation) and *n* was equal to 0.76 ± 0.06 (figure 3*d*). The coefficients of determination *r*^2^ were large, with a mean of 0.85 ± 0.07 across participants, implying a good model fit. This result is consistent with the work on translational slip of Delhaye *et al.* who performed a similar analysis and found a value of *n* of around 0.67 on glass [20] and Barrea *et al.* [38] who found a value of *n* of around 0.66 on Kapton.

The influence of the power law relationship between the coefficient of rotational friction and normal force (figure 3*c*) is reflected in the nonlinear relationship between maximum torque and normal force in figure 3*a*. The larger coefficient of friction at lower normal forces allows for relatively large increases in maximum torque (i.e. traction) with increases in grip force across the force ranges associated with delicate precision manipulation, i.e. 0.5–2 N.

Note that the coefficient of rotational friction, as calculated here, captures two effects. One is the increasing real contact area (cumulative area of all microjunctions made between the fingerprint ridges and the glass), which increases the friction available at each local region of the fingerprint ridges. The second is that the apparent contact area increases in size with the increasing normal force, with more skin coming into contact at the periphery of the contact area (further from the centre of rotation) where it can make a substantial contribution to the maximum torque reached.

### 3.3. Apparent contact area

**Figure 4.**
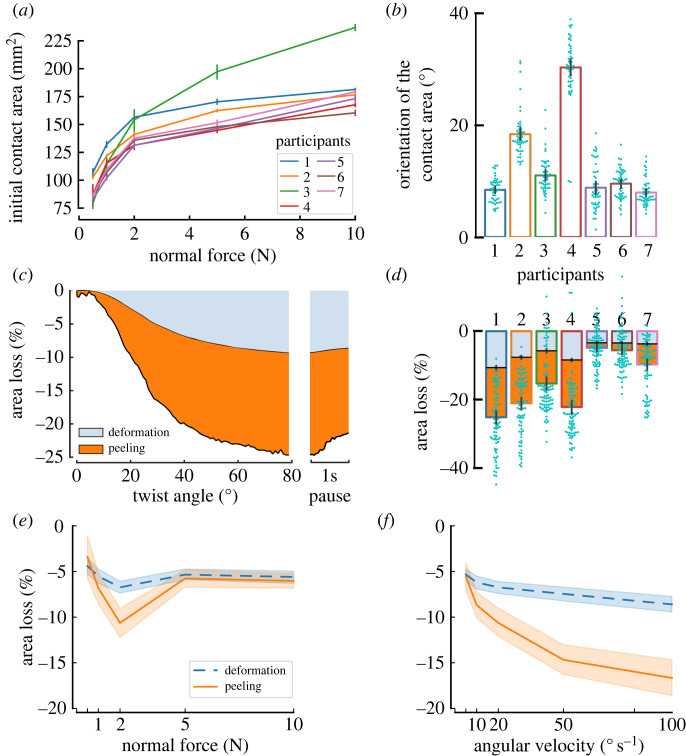
Contact area. (*a*) Contact area after normal loading. (*b*) Change of orientation of the contact area after the 80° rotation, grouped by participant. (*c*) Area reduction attributable to surface deformations and peeling for an example condition and participant (average of five clockwise trials at 2 N and 20° s^−1^); (*d*) relative change between initial contact area after normal loading and contact area after the 80° rotation. Proportion attributable to deformation and peeling are indicated. (*e*,*f*) Averages across participants of the area reduction after completion of the 80° rotation as a function of normal force (*e*) and angular velocity (*f*). Error bars and shaded areas represent 95% confidence intervals.

The apparent contact area after normal loading ($A_{0}^{A}$) varied across participants, likely dependent on their finger morphology and individual biomechanics, as well as on the normal force applied. Indeed, as expected, $A_{0}^{A}$ increased with the normal force (figure 4*a*), with most of the increase in the initial contact area happening at low force levels (between 0.5 N and 2 N) before reaching a plateau. $A_{0}^{A}$ almost doubled between 0.5 N (90.23 ± 11.75 mm^2^) and 10 N (182.22 ± 23.68 mm^2^) across all participants. The impact of the normal force on the $A_{0}^{A}$ is best described by the power law $A_{0}^{A}=aF_{N}^{b}$, where *F*_*N*_ is the normal force, and *a* and *b* are the parameters of the fit. The mean across participants of *a* was 113.59 ± 8.48. The mean across participants of the exponent *b* was 0.21 ± 0.052. The mean of the coefficient of determination *r*^2^ was 0.93 ± 0.032, indicating a good fit. Note that Participant 3 is a clear outlier from the group, achieving larger contact areas than the other six participants at 5 N and 10 N.

This relationship between contact area and normal force for a human finger pad in contact with a planar surface is somewhat consistent with previous works [26,37]. Both Delhaye *et al.* and Dzidek *et al.* obtain power law fit parameter values ranging between 0.36 and 0.52 for *b*. However, some differences are expected, given that those previous studies used a maximum normal force of 2 N, which is far less than the 10 N maximum normal force used in this study. Note also, this power law is the well-known Hertzian model of contact (with a 2/3 exponent), derived from a model of a homogeneous linear elastic sphere in contact with a planar surface [39]. This model is only considered valid for small contact areas relative to the size of the sphere being modelled. The shape and biomechanics of the human finger and the size of the contact area achieved relative to the size of the finger pad violate the assumptions of this Hertzian model and thus we expect some discrepancies between fit and experimental data.

The apparent contact area was affected by the rotation of the stimulus plate. Indeed, both its principal orientation (figure 4*b*) and its size (figure 4*d*) changed with the angular rotation of the plate. Orientation of the contact was significantly affected by normal force (*F* = 5.45, *p* = 0.0029, *η*^2^ = 0.48) without clear trends being visible with no pair of *F*_*N*_ levels reaching significance in the *post hoc* tests, while CW trials led to smaller changes in orientation of the contact area than CCW trials (*F* = 10.82, *p* = 0.017, *η*^2^ = 0.64). The interaction direction–*F*_*N*_ did not reach significance (*F* = 2.13, *p* = 0.15). Change in orientation of the contact area increased with angular velocity (*F* = 24.78, *p* < 0.001, *η*^2^ = 0.70), and this time direction did not reach significance (*F* = 5.23, *p* = 0.062) nor did the interaction (*F* = 1.63, *p* = 0.25). There was a large variability across participants (figure 4*b*).

In most trials (88.83%), the size of the contact area was reduced by the rotation of the plate (figure 4*d*). The reduction in contact area was on average 15±11%. However, at low normal forces, and for only a subset of participants, an increase in the contact area was observed. For those participants showing an increase in contact area, at initial contact, parts of the fingerprint ridges showed little contrast in the captured frames of the video, probably due to the participant having drier skin, which led to a reduced estimation of the contact area size. As moisture increased on the finger pad, the contrast in the image improved, leading to the observed increase in the contact area [40]. For the majority of participants that show a reduction in contact area with rotation, this was driven by two different effects: (i) peeling, i.e. the skin losing contact with the plate, and (ii) deformation, i.e. the triangular elements reducing in size. Peeling accounted for the largest component of the area reduction in most cases (figure 4*c*,*e*,*f*).

The area reduction increased with angular velocity, with a greater increase in peeling than surface deformation (figure 4*e*). The rANOVA showed a significant influence of velocity on both the surface deformation (*F* = 15.46, *p* < 0.001, *η*^2^ = 0.13) and peeling (*F* = 33.53, *p* < 0.001, *η*^2^ = 0.30).

This rate dependence could imply a viscoelastic influence on the skin mechanics to cause more skin to peel from the contact area periphery at higher rates of rotation. A possible explanation is that viscoelastic effects increase the skin’s stiffness. Due to the contact between the plate and the finger being robotically force controlled, if the normal force increased during rotation, due to skin deformation, the plate would be retracted to restore the target force. We observed that the robot retraction movement (vertical movement between the start and the end of the sliding movement) slightly increased with angular velocity, with averages going from 0.08 ± 0.13 mm for 5° s^−1^ to 0.21 ± 0.17 mm for 100° s^−1^, which might contribute to skin peeling as the plate moves away from the finger to maintain the target normal force.

Normal force had no significant influence on the surface deformations component of the reduction in *A*^*A*^ (*F* = 1.32, *p* = 0.29), but affected peeling (*F* = 2.83, *p* = 0.047). Only the pair 1–2 N reached significance in the *post hoc* tests, which should be interpreted with caution due to the order of presentation (see §4.3).

### 3.4. Propagation of the slipping region

As expected, the periphery of the contact area started to slip first. The slip wavefront propagated towards the central region of the contact until full slip occurred (figure 2*b*).

**Figure 5.**
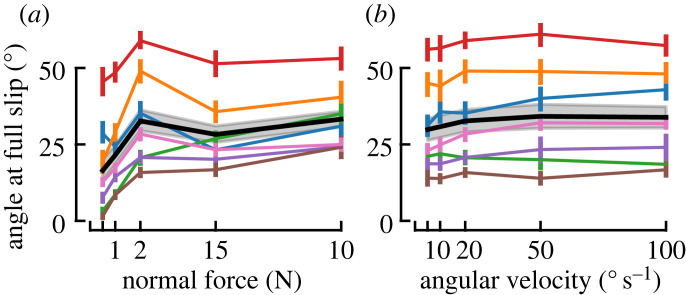
Reaching full slip. Averages (black lines) with 95% confidence intervals (grey shaded areas) across participants of the angle to reach full slip as a function of normal force (*a*) and as a function of angular velocity (*b*). Participant averages are indicated as coloured lines, with 95% confidence intervals.

The twist angle needed to reach full slip increased with normal force (*F* = 14.38, *p* < 0.001, *η*^2^ = 0.19), see figure 5*a*, but only the pair 0.5–10 N reached significance in the *post hoc* tests. Full slip was reached later in CCW than in CW trials (*F* = 8.55, *p* = 0.027, *η*^2^ = 0.0065), and there was no significant interaction between rotation direction and *F*_*N*_ (*F* = 1.34, *p* = 0.28).

Increasing the angular velocity had no practical impact on the rotation angle at full slip (figure 5*b*), which conversely means that it linearly shortened the time available to the participant should they have needed to react before full slip occurred. Although the rANOVA found a significant effect of angular velocity (*F* = 6.01, *p* = 0.0017, *η*^2^ = 0.50), no pairs of *ω* showed significant differences after Bonferroni correction. There were no significant effects of direction (*F* = 2.70, *p* = 0.15), nor interaction (*F* = 1.89, *p* = 0.14).

### 3.5. Local strains

The central portion of the contact area sticks to the plate and moves as a rigid-body rotation, while skin in the periphery slips, slows down and then stops. Surface strains thus arise behind the slip wavefront.

Aggregating local skin deformations across the finger pad, for all subjects, and over the time course of all trials into summary heat maps risks obfuscating the rich patterns that emerge throughout the slipping duration. We observe very similar patterns across all participants, which differ mostly in the times at which different amounts of slip have occurred, the contact area size after normal force loading, the amount of rotation of the contact area throughout the trial or the intensity of the deformations experienced on the slip wavefront as it propagates. Therefore, we provide some representative examples in figure 7 and comparisons to help illustrate these patterns for the reader. In addition, we used the median maximum shear over the contact area |*e*_*s*,end_| to allow quantitative comparisons between conditions.

#### 3.5.1. Principal strains and maximum shear

**Figure 6.**
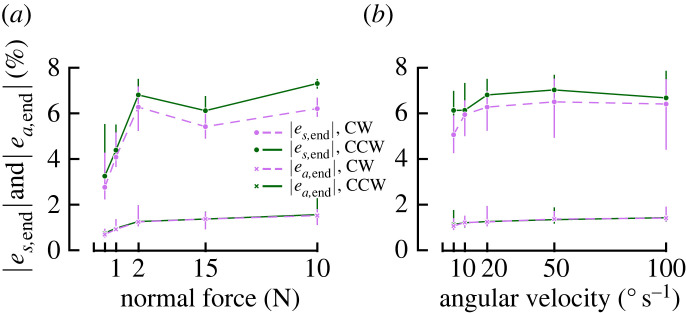
Strains invariants. Averages with 95% confidence intervals across participants of the median shear strain (|*e*_*s*,end_|) and the area invariant (|*e*_*a*,total_|) over the contact area after the full rotation as a function of normal force (*a*) and as a function of angular velocity (*b*).

**Figure 7.**
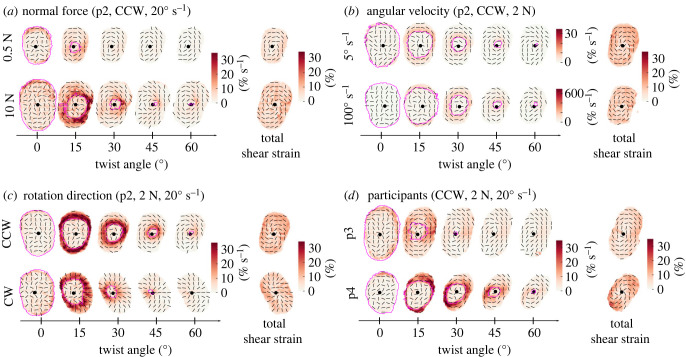
Illustrative maximum shear across conditions. The centre of rotation is indicated by a black dot, the slip wavefront by a magenta contour and the maximum shear strain’s orientation by black lines. (*a*) *Comparison of different normal forces, at 20° s^−1^.* Comparison of maximum shear across finger pad skin for a normal force of 0.5 N versus 10 N, for a counterclockwise trial with a rotation rate of 20° s^−1^ (Participant 2). The heat map across each finger pad contact area illustrates the maximum shear rate at each skin location. The last column shows the total of the maximum shear for the full 80° rotation. As expected, the contact area is larger for the larger normal force. More slip happens earlier at the smaller normal force. (*b*) *Comparison of different angular velocities, at 2 N.* Comparison of maximum shear across finger pad skin for an angular velocity of 5° s^−1^ versus 100° s^−1^, for a counterclockwise trial with a normal force of 2 N (Participant 2). Since the rates of rotation differ by a factor of 20, for visualization purposes, we make the scale bar ranges also differ by this factor. Despite the large difference in the rotation rate between the two trials, there is very little difference in the spatial and temporal patterns of maximum shear rate, aside from their differing magnitudes. (*c*) *Comparison of rotation direction, at 2 N, 20° s^−1^ for Participant 2.* (*d*) *Comparison of two participants, at 2 N, 20° s^−1^.* An illustrative comparison of the time course of single trials taken from two different participants. We observe that, while the overall pattern of the time series is generally similar across these two (and all) participants, the timing of events does vary between participants; e.g. Participant 3 (p3) experiences a lot of slip early in the rotation, whereas Participant 4 (p4) achieves the same amount of slip at a later time (and larger angle of rotation).

In addition to increasing $A_{0}^{A}$ and delaying full slip, increasing the *F*_*N*_ also increased local strain rates (figure 7*a*). This is also visible as a significant influence of *F*_*N*_ on |*e*_*s*,end_| (*F* = 24.53, *p* < 0.001, *η*^2^ = 0.26), as shown in figure 6*a*.

Angular velocity greatly influenced the strain rates expressed in % s^−1^ (note the different scales in the two rows of figure 7*b*). However, it did not have a strong effect on strain rates in $\text{\%}{/}^{\circ }$, meaning that the total strain after the full rotation was completed did not strongly differ between levels of *ω* (figure 6*b*). Despite reaching statistical significance, *ω* only showed a small effect size on |*e*_*s*_| (*F* = 3.55, *p* = 0.021, *η*^2^ = 0.01).

CCW trials resulted in slightly higher shear strains than CW trials (figure 6), and mirrored shear patterns (figure 7*c*). Although it reached significance in both rANOVAs testing the effect of *F*_*N*_ and *ω* on |*e*_*s*_|, the effect size was small in both cases (*F*_*N*_: *p* = 0.0042, *η*^2^ = 0.02 and *ω*: *p* = 0.015, *η*^2^ = 0.01).

$A_{0}^{A}$, twist angle at full slip, local strain rates and total strains all varied across participants, as illustrated in figure 7*d*.

#### 3.5.2. Strain energy

**Figure 8.**
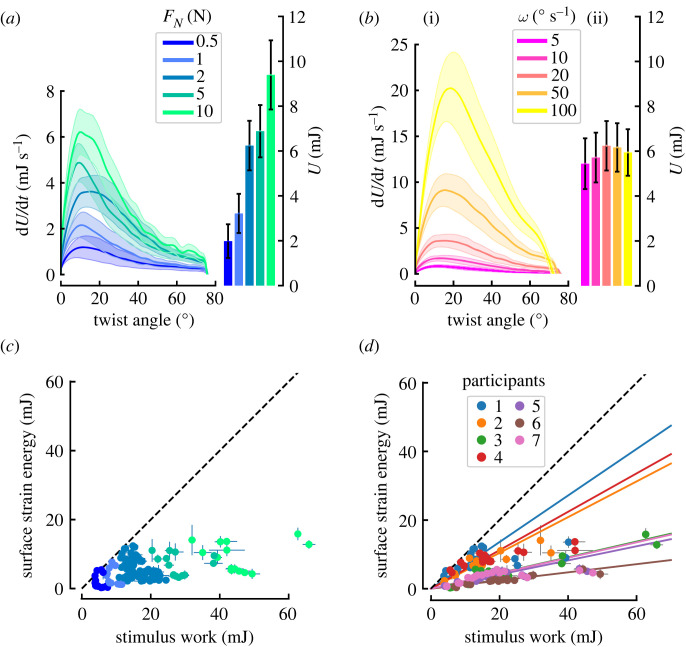
Evolution of strain energy. The influence of normal force (*a*) and angular velocity (*b*) on the evolution of the total strain energy rate (d*U*/d*t*) with respect to stimulus displacement (line graphs) and final total strain energy (bar charts). Each trace and box shows the mean across subjects. Shaded areas and bars show standard error. Note that in (*b*), the higher the value of *ω*, the less time the rotation lasts, which is why despite having higher energy rates fast angular velocities do not lead to higher time integrals. *Surface strain energy versus external work*. The work provided by the stimulus is the upper limit on surface deformation energy (dashed line). Only the colour code changes between (*c*) and (*d*). Note that the surface strain energy was estimated from the first experiment, while the external work was computed from the second experiment. Each data point was computed as the average value of the five repeats of the trials corresponding to a single condition. (*c*) Normal force increases the stimulus work as well as the surface deformation energy. (*d*) When colour coding by participants, it becomes apparent that the proportion of the available work converted into surface deformation decreases with the increasing normal force. Linear fits constrained to pass through the origin do not fit the data very well for participants with large deformation.

Both the maximum strain energy rate (d*U*/d*t*) and the total strain energy (*U*) (*F* = 22.39, *p* < 0.001, *η*^2^=0.79) increased with increasing normal force (figure 8*a*). This is expected for two reasons. Firstly, a larger normal force will result in a larger *A*^*A*^, and so the opportunity for more skin to experience deformation (figure 4). Secondly, a larger normal force will provide greater traction at any local region of skin, and so the propensity to generate more extreme deformations of the skin; this is evidenced by increasing maximum torque with increasing normal force (see figure 3*a*).

Although the maximum strain energy rate (d*U*/d*t*) increased with angular velocity, the total strain energy (*U*) did not (*F* = 1.46, *p* = 0.24) (figure 8*b*(ii)). Indeed, for larger angular velocities, the strain energy peak increased, whereas the duration of the rotation was reduced in proportion, leading to similar total strain energy.

The surface strain energy was compared to the external work applied to the finger by the stimulus (i.e. integral of torque-angle product over time). Note that the surface strain energy was estimated from the first experiment, while external work was computed from the second experiment. The proportion of work used to deform the skin reduced with normal force for the participants undergoing the most surface deformation (note how the larger work points deviate from the linear fits for Participants 1, 2 and 4 in figure 8*d*).

It can be noted that as normal force increases, more external work is done by the stimulus and more strain energy deforms the skin. However, as the normal force increased, a smaller fraction of the available work was converted into surface deformation (figure 8*c*). Excess energy could be converted into deformation of the skin bulk, and some into heat. It should be noted that differences across subjects are most probably related to differences in skin properties (e.g. stiffness), which are set constant in the calculation.

## 4. Discussion

We studied the effects of a torsional load on the finger pad of human participants by pressing a glass plate against their skin at a robotically controlled normal force, then rotating it at a constant angular velocity. As the stimulus plate rotated, we observed partial slippage of the skin in contact with the plate. The periphery of the contact area started slipping first, while the region closer to the centre of rotation stuck to the stimulus plate and moved as a rigid body. While this incipient slip pattern has been well documented for translational movements, this is the first study to comprehensively describe the finger pad skin mechanics under torsional stimulation. We observed that at the slip wavefront, an annulus of strains dominated by shear is present. While there are differences in the magnitudes and timeline of skin deformations between participants, trials generally follow a similar pattern for all participants across all experimental conditions.

### 4.1. Effect of force and velocity

We tested a wide (20-fold) range of angular velocities and normal forces, using values relevant to both object manipulation and tactile exploration. We observed a significant increase in torque with both normal force and angular velocity. The effect of normal force was expected from the Coulomb model of friction (*F*_*T*_ = *μF*_*N*_). Indeed, an increasing normal force deforms the finger pad skin, thus increasing both the apparent contact area *A*^*A*^ and also the real contact area (number and strength of atomic/molecular bonds giving rise to friction forces, which is a subset of the visible fingerprint ridge contact area [36]). With the increase in contact area, the amount of traction available will increase and lead to a higher torque reached at full slip. However, the fact that the increase in torque with *F*_*N*_ is sub-linear (also evidenced by the decrease of *μ*_rot_ with normal force) highlights the limited validity of the Coulomb model for the finger pad.

Maximum torque is also affected by angular velocity, with small increases in maximum torque achieved for an increase in angular velocity (figure 3*b*).

### 4.2. Comparison between rotating and translating stimuli

The present study extends the work done using rectilinear stimuli [20,26]. In both cases, partial slippage of the contact area was observed, starting at its periphery. The skin at the centre of the contact area slips last, while surface strains are present at the slip wavefront. This similarity between slips under rotation and translation was expected due to the curvature of the finger pad, resulting in smaller traction at the periphery of the contact area, which is where slips first occur when the contact is challenged by shear forces.

Despite this similarity, there are differences in strain patterns between rectilinear and torsional motion. In rectilinear motion, there is compression at the leading edge and dilation at the trailing edge, accompanied by net change in area locally. In rotation, compressive and dilative principal strains, which are similar in magnitude, give rise to a strong shearing effect within the incipient slip wavefront annulus, but only small area changes. Although their patterns differ, strains in both types of motion contain information regarding the safety of the contact.

The present study proposes maximum shear as a tool to study surface skin deformations. We showed that it is highly present in rotations. It has the advantage of being an invariant of the strain tensor, bypassing the need to define a fixed coordinate system, which is not a trivial task when studying complex motions, be they passive rotations or combinations of translations and rotations encountered during natural object manipulation.

### 4.3. Limitations and sources of error

#### 4.3.1. Linking torque and deformations

Because of the noisy recording of torque during experiment 1, torque had to be acquired during a second experiment. This limits the possibility of precisely linking time points in the torque evolution with skin deformations and contact area reductions. Indeed, as the experiments were conducted on separate days, parameters such as the moisture of the participant’s finger could have impacted the friction. However, given that both experiments were performed with the same participants and the same experimental equipment, very similar variations in the behaviour of their fingers were achieved, hence allowing us to qualitatively compare the results of experiment 1 and experiment 2. One possible way to circumvent this issue would be to use a ring-shaped force sensor, rather than a disc, allowing the finger pad to be imaged through the hole in the ring-shaped sensor.

#### 4.3.2. Effect of the order of presentation

Data points at 2 N do not always fit the general trends, such as for area loss (figure 4*e*), the twist angle at full slip (figure 5*a*), or the strain energy (figure 8*a*). The order of presentation of the trials at different normal forces was not randomized, as the focus of the camera needed to be adjusted for each normal force. The trials were presented in blocks of similar normal force in the following order: 0.5 N, 1 N, 5 N, 10 N and 2 N. Two effects are at play: change of normal force and total experiment time. The latter might have an influence on finger characteristics, such as moisture increasing with occlusion time [41]. In hindsight, this effect could have been mitigated by changing the presentation order across participants.

### 4.4. Potential implications for object manipulation

Trial-to-trial adaptation of grip force is slower in the presence of torque, possibly explained by differences in cutaneous feedback received [42]. In the present study, we confirm that local deformation patterns are qualitatively different under torsion than translation. In addition, for similar minimum required grip forces, there might be more partial slips in the presence of torque, as towards the periphery of the contact area, there is not only a decrease in pressure (hence traction) due to the curvature of the finger pad but also an increase in tangential force due to torsion. Devising an active manipulation experimental paradigm comprising both torque and imaging of cutaneous deformations could help investigate whether the amount of partial slips and/or the nature of the strains (shear versus area change) created affect grip force adaptation.

### 4.5. Variability between participants

An important variation in the biomechanical response of the skin was observed across subjects. A typical example of such variation concerns the angle needed to reach full slip: figure 5 shows that there is almost a four-fold difference across participants in the twist angle required to reach full slip (e.g. at 2 N and 20° s^−1^, subject averages ranged from 15.81° to 58.93°). The consequence of this, given that the plate rotates at a constant angular velocity, is that the time to full slip varies between participants. In addition, figure 7*d* provides an illustrative comparison of slip propagation for Participant 3 (p3) and Participant 4 (p4). It shows the difference in the timing of the strains’ evolution and highlights the difference in strain magnitude experienced by participants. This wide range of responses of the skin to a single stimulus was also pointed out by Wang & Hayward [43] and illustrates the need to pay attention to biomechanical variables when searching for the causes of sensory afferent responses, instead of focusing solely on gross mechanical stimulation parameters, such as the time course of tangential force and torque, or velocity, or stimulus curvature [15,29,44,45]. Although some afferent types (slowly adapting) are sensitive to the resulting force vector, others (fast adapting type I) might correlate better with local deformations [21]. It is also an argument against the use of standardized fingers when reporting data from multiple participants. Two afferents reported at the same location on a standardized finger might be subjected to very different strains, even if the external stimulus was well controlled. Ultimately, afferents respond to local events that trigger their end-organs, and ignoring the variation in skin dynamics between subjects might blur subsequent analyses. Linking observed events as close as possible to the receptive fields of afferents, such as surface skin deformation, can mitigate this effect. In addition, statistical tools, such as linear mixed effect models, which take into account the nested structure of data, could prove useful when relating microneurography recordings with mechanical variables [46]. It is likely that other factors, such as the orientation and depth of an afferent in the skin, will also lead to different responses from afferents of the same type. Hence, avoiding the use of standardized fingers in the analysis of afferent response will improve the understanding of the differences in afferent behaviour but will not totally explain these differences.

### 4.6. Potential links with mechanoreceptor firing

For rectilinear motion, surface strains have been linked to afferent firing [21,47], and it is likely that a similar link exists in rotation that further microneurography and/or psychophysics studies would highlight. Furthermore, microneurography studies with a broader variety of mechanical stimuli might help pinpoint which aspects of the skin deformation cause afferents to fire. For example, an experiment with rotation, as in this article, would not allow for the determination of whether compression, dilation and shear, which are all simultaneously present on the slip wavefront, are primarily responsible for afferent firing. As highlighted earlier, translating stimuli show more area changes and less shearing than rotating stimuli, and thus it might be possible to use a paradigm involving both stimuli types to disentangle the sensitivity of the afferents under investigation to specific strains.

In the current study, we highlighted skin peeling at the periphery of the contact area, and some mechanoreceptors might be sensitive to that. SA-I afferents are typically described as sensitive to indentation [5] and might respond to the skin losing contact with the plate. A limitation, however, is that with experiments measuring surface deformation only, we do not have information about the deformation of the bulk of the finger pad in which the sensory afferents are embedded, or other locations outside the contact area such as the nail bed. Alternative techniques to study skin deformation include optical coherence tomography [48], multi-camera systems [18] or computational modelling such as finite element modelling [49,50].

## 5. Conclusion

In this work, we showed the complex skin deformations at the interface between the human finger pad and a glass plate under a torsional load. We highlighted the important shear effects at the periphery of the contact, between the stuck and slipping parts of the skin. These mechanics are important as they play a significant role in generating the tactile information used during manipulation. We further show that the mechanics of the skin vary widely across participants, hence highlighting the importance of accounting for such variability when ensembling the results of neurological recordings from multiple participants onto a standard finger. In future work, we would like to combine microneurographic recordings with this imaging approach and a novel paradigm involving both translation and rotation, to better understand how different strain types (dilation, compression and shear) contribute to the responses of different afferent types and hence our ability to sense grip security. We will also consider other imaging modalities which allow us to measure skin deformation outside of the contact area between the skin and the plate, such as at the sides or in the bulk of the finger.

## Data Availability

The raw forces and platform kinematics files; 1DxTime variables extracted from the videos such as displayed in figure 2*a*; and 1D summary variables over trials (e.g. the angle at full slip for each trial) are available at: https://zenodo.org/record/7646119#.Y-5YWXbP1D8 [51]. Jupyter Notebooks used for statistical analysis and corresponding figures are available at: https://bitbucket.org/s_dubois/jupyternotebooks/src/master/.

## References

[1] Augurelle AS, Smith AM, Lejeune T, Thonnard JL. 2003 Importance of cutaneous feedback in maintaining a secure grip during manipulation of hand-held objects. J. Neurophysiol. **89**, 665-671. (10.1152/jn.00249.2002) 12574444

[2] Witney AG, Wing A, Thonnard JL, Smith AM. 2004 The cutaneous contribution to adaptive precision grip. Trends Neurosci. **10**, 637-643. (10.1016/j.tins.2004.08.006) 15374677

[3] Park J, Son B, Han I, Lee W. 2020 Effect of cutaneous feedback on the perception of virtual object weight during manipulation. Sci. Rep. **10**, 1357. (10.1038/s41598-020-58247-5) 31992799 PMC6987230

[4] Corniani G, Saal HP. 2020 Tactile innervation densities across the whole body. J. Neurophysiol. **124**, 1229-1240. (10.1152/jn.00313.2020) 32965159

[5] Handler A, Ginty DD. 2021 The mechanosensory neurons of touch and their mechanisms of activation. Nat. Rev. Neurosci. **22**, 521-537. (10.1038/s41583-021-00489-x) 34312536 PMC8485761

[6] Barrea A, Delhaye BP, Lefèvre P, Thonnard JL. 2018 Perception of partial slips under tangential loading of the fingertip. Sci. Rep. **8**, 7032. (10.1038/s41598-018-25226-w) 29728576 PMC5935679

[7] Willemet L, Kanzari K, Monnoyer J, Birznieks I, Wiertlewski M. 2021 Initial contact shapes the perception of friction. Proc. Natl Acad. Sci. USA **118**, e2109109118. (10.1073/pnas.2109109118) 34857635 PMC8670444

[8] Johansson RS, Flanagan JR. 2009 Coding and use of tactile signals from the fingertips in object manipulation tasks. Nat. Rev. Neurosci. **10**, 345-359. (10.1038/nrn2621) 19352402

[9] Saal HP, Bensmaia SJ. 2014 Touch is a team effort: interplay of submodalities in cutaneous sensibility. Trends Neurosci. **37**, 689-697. (10.1016/j.tins.2014.08.012) 25257208

[10] Corniani G, Casal MA, Panzeri S, Saal HP. 2022 Population coding strategies in human tactile afferents. PLoS Comput. Biol. **18**, e1010763. (10.1371/journal.pcbi.1010763) 36477028 PMC9762576

[11] Khamis H, Birznieks I, Redmond SJ. 2015 Decoding tactile afferent activity to obtain an estimate of instantaneous force and torque applied to the fingerpad. J. Neurophysiol. **114**, 474-484. (10.1152/jn.00040.2015) 25948866 PMC4509394

[12] Delhaye BP, Xia X, Bensmaia SJ. 2019 Rapid geometric feature signaling in the simulated spiking activity of a complete population of tactile nerve fibers. J. Neurophysiol. **121**, 2071-2082. (10.1152/jn.00002.2019) 30943102 PMC6620699

[13] Weber AI, Saal HP, Lieber JD, Cheng JW, Manfredi LR, Dammann III JF, Bensmaia SJ. 2013 Spatial and temporal codes mediate the tactile perception of natural textures. Proc. Natl Acad. Sci. USA **110**, 17 107-17 112. (10.1073/pnas.1305509110) PMC3800989 24082087

[14] Cadoret G, Smith AM. 1996 Friction, not texture, dictates grip forces used during object manipulation. J. Neurophysiol. **75**, 1963-1969. (10.1152/jn.1996.75.5.1963) 8734595

[15] Loutit AJ, Wheat HE, Khamis H, Vickery RM, Macefield VG, Birznieks I. 2022 How tactile afferents in the human fingerpad encode tangential torques associated with manipulation: are we better than monkeys? *bioRxiv*. (10.1101/2022.07.11.499647) PMC10254986 37142429

[16] Saal HP, Delhaye BP, Rayhaun BC, Bensmaia SJ. 2017 Simulating tactile signals from the whole hand with millisecond precision. Proc. Natl Acad. Sci. USA **114**, E5693-E5702. (10.1073/pnas.1704856114) 28652360 PMC5514748

[17] Birznieks I, Macefield VG, Westling G, Johansson RS. 2009 Slowly adapting mechanoreceptors in the borders of the human fingernail encode fingertip forces. J. Neurosci. **29**, 9370-9379. (10.1523/JNEUROSCI.0143-09.2009) 19625527 PMC6665555

[18] Kao AR, Xu C, Gerling GJ. 2022 Using digital image correlation to quantify skin deformation with Von Frey monofilaments. IEEE Trans. Haptic **15**, 26-31. (10.1109/TOH.2021.3138350) PMC9006180 34951855

[19] André T, Lévesque V, Hayward V, Lefèvre P, Thonnard JL. 2011 Effect of skin hydration on the dynamics of fingertip gripping contact. J. R. Soc. Interface **8**, 1574-1583. (10.1098/rsif.2011.0086) 21490002 PMC3177614

[20] Delhaye BP, Barrea A, Edin BB, Lefèvre P, Thonnard JL. 2016 Surface strain measurements of fingertip skin under shearing. J. R. Soc. Interface **13**, 20150874. (10.1098/rsif.2015.0874) 26888949 PMC4780562

[21] Delhaye BP, Jarocka E, Barrea A, Thonnard JL, Edin BB, Lefèvre P. 2021 High-resolution imaging of skin deformation shows that afferents from human fingertips signal slip onset. eLife **10**, e64679. (10.7554/eLife.64679) 33884951 PMC8169108

[22] Johansson RS, Westling G. 1987 Signals in tactile afferents from the fingers eliciting adaptive motor responses during precision grip. Exp. Brain Res. **66**, 141-154. (10.1007/BF00236210) 3582528

[23] Schiltz F, Delhaye BP, Crevecoeur F, Thonnard JL, Lefèvre P. 2021 Fast grip force adaptation to friction relies on localized fingerpad strains. *bioRxiv*. (10.1101/2021.07.20.452911) PMC10793957 38232162

[24] Levesque V, Hayward V. 2003 Experimental evidence of lateral skin strain during tactile exploration. In *Proc. Eurohaptics 2003, Dublin, Ireland, 6–9 July 2003*, pp. 261-275.

[25] Tada M, Kanade T. 2004 An imaging system of incipient slip for modeling how human perceives slip of a fingertip. In *Annual Int. Conf. of IEEE Engineering in Medicine and Biology Society, San Francisco, CA, USA, 1–5 September 2004*, pp. 2045-2048. (10.1109/IEMBS.2004.1403601) 17272121

[26] Delhaye BP, Lefèvre P, Thonnard JL. 2014 Dynamics of fingertip contact during the onset of tangential slip. J. R. Soc. Interface **11**, 20140698. (10.1098/rsif.2014.0698) 25253033 PMC4191101

[27] Kinoshita H, Bäckström L, Flanagan JR, Johansson RS. 1997 Tangential torque effects on the control of grip forces when holding objects with a precision grip. J. Neurophysiol. **78**, 1619-1630. (10.1152/jn.1997.78.3.1619) 9310447

[28] Goodwin AW, Jenmalm P, Johansson RS. 1998 Control of grip force when tilting objects: effect of curvature of grasped surfaces and applied tangential torque. J. Neurosci. **18**, 10 724-10 734. (10.1523/JNEUROSCI.18-24-10724.1998) 9852607 PMC6793331

[29] Birznieks I, Wheat HE, Redmond SJ, Salo LM, Lovell NH, Goodwin AW. 2010 Encoding of tangential torque in responses of tactile afferent fibres innervating the fingerpad of the monkey. J. Physiol. **588**, 1057-1072. (10.1113/jphysiol.2009.185314) 20142274 PMC2852995

[30] André T, Lefèvre P, Thonnard JL. 2009 A continuous measure of fingertip friction during precision grip. J. Neurosci. Methods **179**, 224-229. (10.1016/j.jneumeth.2009.01.031) 19428531

[31] Moore RT. 1989 An analysis of ridge-to-ridge distance on fingerprints. J. Forensic. Ident. **39**, 231-238.

[32] Lucas BD, Kanade T. 1981 An iterative image restoration technique with an application to stereo vision. In *Proc. Int. Conf. on Artificial Intelligence (IJCAI), Vancouver, Canada, 24–28 August 1981*, vol. 2, pp. 674–679.

[33] Shi J, Tomasi C. 1994 Good features to track. In *Proc. IEEE Conf. on Computer Vision and Pattern Recognition, Seattle, WA, USA, 21–23 June 1994*, pp. 593–600. (10.1109/CVPR.1994.323794)

[34] Bouguet JY. 2001 Pyramidal implementation of the Lucas Kanade feature tracker description of the algorithm. Hillsboro, OR: Intel Corporation, Microprocessor Research Labs.

[35] Lengiewicz J, de Souza M, Lahmar MA, Courbon C, Dalmas D, Stupkiewicz S, Scheibert J. 2020 Finite deformations govern the anisotropic shear-induced area reduction of soft elastic contacts. J. Mech. Phys. Solids **143**, 104056. (10.1016/j.jmps.2020.104056)

[36] Sahli R, Pallares G, Ducottet C, Ben Ali IE, Al Akhrass S, Guibert M, Scheibert J. 2018 Evolution of real contact area under shear and the value of static friction of soft materials. Proc. Natl Acad. Sci. USA **115**, 471-476. (10.1073/pnas.1706434115) 29295925 PMC5776957

[37] Dzidek BM, Adams MJ, Andrews JW, Zhang Z, Johnson SA. 2017 Contact mechanics of the human finger pad under compressive loads. J. R. Soc. Interface **14**, 20160935. (10.1098/rsif.2016.0935) 28179549 PMC5332579

[38] Barrea A, Cordova Bulens D, Lefevre P, Thonnard JL. 2016 Simple and reliable method to estimate the fingertip static coefficient of friction in precision grip. IEEE Trans. Haptic **9**, 492-498. (10.1109/TOH.2016.2609921) 27831889

[39] Greenwood JA. 1985 Formulas for moderately elliptical hertzian contacts. J. Tribol. **107**, 501-504. (10.1115/1.3261116)

[40] Dzidek B, Bochereau S, Johnson SA, Hayward V, Adams MJ. 2017 Why pens have rubbery grips. Proc. Natl Acad. Sci. USA **114**, 10 864-10 869. (10.1073/pnas.1706233114) PMC5642691 28973874

[41] Pasumarty SM, Johnson SA, Watson SA, Adams MJ. 2011 Friction of the human finger pad: influence of moisture, occlusion and velocity. Tribol. Lett. **44**, 117-137. (10.1007/s11249-011-9828-0)

[42] Crevecoeur F, Giard T, Thonnard JL, Lefèvre P. 2011 Adaptive control of grip force to compensate for static and dynamic torques during object manipulation. J. Neurophysiol. **106**, 2973-2981. (10.1152/jn.00367.2011) 21940610

[43] Wang Q, Hayward V. 2007 In vivo biomechanics of the fingerpad skin under local tangential traction. J. Biomech. **40**, 851-860. (10.1016/j.jbiomech.2006.03.004) 16682045

[44] Birznieks I, Jenmalm P, Goodwin AW, Johansson RS. 2001 Encoding of direction of fingertip forces by human tactile afferents. J. Neurosci. **21**, 8222-8237. (10.1523/JNEUROSCI.21-20-08222.2001) 11588194 PMC6763843

[45] Goodwin AW, Browning AS, Wheat HE. 1995 Representation of curved surfaces in responses of mechanoreceptive afferent fibers innervating the monkey’s fingerpad. J. Neurosci. **15**, 798-810. (10.1523/JNEUROSCI.15-01-00798.1995) 7823181 PMC6578298

[46] Yu Z, Guindani M, Grieco SF, Chen L, Holmes TC, Xu X. 2022 Beyond t test and ANOVA: applications of mixed-effects models for more rigorous statistical analysis in neuroscience research. Neuron **110**, 21-35. (10.1016/j.neuron.2021.10.030) 34784504 PMC8763600

[47] Edin BB. 2004 Quantitative analyses of dynamic strain sensitivity in human skin mechanoreceptors. J. Neurophysiol. **92**, 3233-3243. (10.1152/jn.00628.2004) 15548636

[48] Lee ZS, Maiti R, Carré MJ, Lewis R. 2020 Morphology of a human finger pad during sliding against a grooved plate: a pilot study. Biotribology **21**, 100114. (10.1016/j.biotri.2019.100114)

[49] Dandekar K, Raju BI, Srinivasan MA. 2003 3-D finite-element models of human and monkey fingertips to investigate the mechanics of tactile sense. J. Biomech. Eng. **125**, 682-691. (10.1115/1.1613673) 14618927

[50] Wang Y, Baba Y, Lumpkin EA, Gerling GJ. 2016 Computational modeling indicates that surface pressure can be reliably conveyed to tactile receptors even amidst changes in skin mechanics. J. Neurophysiol. **116**, 218-228. (10.1152/jn.00624.2015) 27098029 PMC4961760

[51] du Bois de Dunilac S, Córdova Bulens D, Lefèvre P, Redmond SJ, Delhaye BP. 2023 Biomechanics of the finger pad in response to torsion [Data set]. Zenodo. (10.5281/zenodo.7646119) PMC10113816 37073518

